# Antibiotic-exposure guided prevalence screening for vancomycin-resistant Enterococcus: better value for hospitals

**DOI:** 10.1017/ice.2025.10332

**Published:** 2026-02

**Authors:** Victoria Williams, Jonah Chevrier, Marion Elligsen, Philip W. Lam, Robert Kozak, Jeff E. Powis, Jerome A. Leis

**Affiliations:** 1 Sunnybrook Health Sciences Centre, Toronto, ON, Canada; 2 Michael Garron Hospital, Toronto, ON, Canada; 3 Temerty Faculty of Medicine, University of Toronto, Toronto, ON, Canada; 4 Centre for Quality Improvement and Patient Safety, University of Toronto, Toronto, ON, Canada

Prevalence screening for antibiotic-resistant organisms including vancomycin-resistant enterococci (VRE) is recommended on hospital units at high-risk of acquisition or where transmission is suspected.^1,2^ Antibiotic exposure is a well-recognized risk factor of VRE acquisition yet not routinely incorporated into decisions about which patients should be screened.^3–5^ We hypothesized that more selective prevalence screening based on antibiotic exposure could reduce the number of screening tests collected without diminishing VRE detection.

We performed a multicenter retrospective cohort study of all acute-care inpatients screened for VRE as part of point prevalence surveys between January 1 and December 31 2024 at two acute-care teaching hospitals served by the same microbiology laboratory. Hospital A is a 515-bed community teaching hospital and hospital B is a 717-bed university-affiliated trauma hospital. VRE screening for reasons other than point prevalence surveys (eg, admission screening, contact tracing) were excluded, as were patients admitted for less than a calendar day or already known to be VRE colonized.

Patients were classified into two cohorts based on minimum of one-dose of antibiotics, including surgical prophylaxis, received within the preceding 30 days: High-risk (3^rd^ generation cephalosporins, β-lactam/β-lactamase inhibitors, fluoroquinolones, carbapenems and glycopeptides), and low-risk (all other antibiotics on the hospitals’ formulary/no antibiotics). High-risk antibiotics were selected a priori based on their antibacterial spectrum and known association with VRE acquisition.^6^ The primary outcome was detection of VRE from a rectal swab obtained as part of unit point prevalence screening. Prevalence screening was conducted for three purposes: following detection of a new case of healthcare-acquired VRE attributed to the unit (HA), as part of an outbreak investigation (OB) and routine screening on high-risk units (HR). Percent positivity for VRE prevalence screening and performance characteristics (sensitivity, specificity, positive predictive value, negative predictive value) were calculated for patients with high-risk antibiotic exposures and those receiving any antibiotic. Research ethics board approval was obtained.

Between January 1 and December 31 2024, 1675 VRE eligible point prevalence screening specimens were obtained including 351 (21.0%) from hospital A and 1324 (79.0%) from hospital B. These included 767 (45.8%) HA, 714 (42.6%) OB, and 194 (11.6%) HR prevalence screens. Overall percent positivity for VRE was 2.6% (43/1675) including 4.0% (14/351) and 2.2% (29/1324) at hospital A and B, respectively. In the 30 days preceding screening, 1071 (63.9%) patients received any antibiotic including 42.7% at hospital A and 69.6% at hospital B (*p* < 0.001). Overall, there were 762 (48.2%) patients who received a high-risk antibiotic including 124 (35.3%) at hospital A and 638(48.2%) at hospital B (*p* < 0.001). High-risk antibiotic exposure by class was 452 (27.0%) β-lactam/β-lactamase inhibitors, 358 (21.4%) third generation cephalosporins, 177 (10.6%) glycopeptides, 128 (7.6%) fluoroquinolones, and 113 (6.7%) carbapenems. The most frequently received low-risk antibiotic was cefazolin (26.3%), followed by metronidazole (4.7%), cephalexin (4.3%), and azithromycin (3.9%).

All patients with VRE detected during point prevalence screening had received a high-risk antibiotic. Among patients that received a high-risk antibiotic the VRE positivity was 5.6% (43/762). By prevalence screening type, the percent positivity among patients on high-risk antibiotics was 7.5% (26/347) for HA, 3.6% (12/338) for OB, and 6.5% (5/77) for HR screens. Performance characteristics of VRE point prevalence screening based on antibiotic exposure are presented in Table 1. Exposure to high-risk antibiotics was associated with better positive predictive value (PPV) than low-risk or any antibiotic, while maintaining negative predictive value (NPV) of 100% (95% CI, 99.6–100).

**Table 1.** Performance characteristics of VRE point prevalence screening based on exposure to antibiotics as opposed to universal screening (*n* = 1675)

Parameter	Value (%)	95% CI
**Any antibiotic^^^**		
Sensitivity	100	91.8, 100
Specificity	37.0	34.7, 39.4
Positive predictive value	4.0	3.0, 5.4
Negative predictive value	100	99.4, 100
High-risk antibiotic*		
Sensitivity	100	91.8, 100
Specificity	55.9	53.5, 58.3
Positive predictive value	5.6	4.2, 7.5
Negative predictive value	100	99.6, 100
**Low-risk antibiotic**		
Sensitivity	0	0.0, 1.2
Specificity	96.9	95.8, 97.7
Positive predictive value	0	0.0, 8.2
Negative predictive value	81.1	79.1, 80.9

^ Includes both high and low-risk antibiotics; *includes 3^rd^ generation cephalosporins, β-lactam/β-lactamase inhibitors, fluoroquinolones, carbapenems and glycopeptides; CI = confidence interval.

Implementing antibiotic-exposure guided prevalence screening for VRE would have avoided over 50% of prevalence screening tests collected at our hospitals, equivalent to 913 tests over the span of one year, without missing a VRE acquisition. This finding was irrespective of whether or not the prevalence screen was performed on a unit with suspected VRE transmission.

The high-risk antibiotics selected in our study are known to facilitate the acquisition of VRE by disturbing the microbiome and depleting Gram-negative commensals that provide a protective barrier to overgrowth of enterococci and replacement of sensitive strains with VRE.^6^ Despite this evidence, antibiotic history has not been routinely incorporated into risk assessments likely due to uncertainty about whether antibiotic history is sensitive enough to minimize risk of missing VRE acquisitions particularly during outbreaks.

Previous studies of prevalence screening performed among all inpatients reported percent positivity ranging from 0.4% to 5.8%.^7,8^ We found similar overall percent positivity of 2.6%, with over doubling of yield to 5.6% using antibiotic-exposure guided screening while maintaining sensitivity. The improved value of VRE prevalence screening for hospitals using this approach would be slightly offset by an increase in the workload of the Infection Preventionists needing to review antibiotic history. To minimize workload, antibiotic-guided screening could be automated into health information systems upon ordering VRE prevalence screens.

Some hospitals do not routinely screen for VRE despite evidence suggesting that it is associated with lower risk of healthcare-associated bacteremia.^9^ A key barrier to VRE screening is cost, which could be mitigated using an antibiotic-guided approach.

A strength of our study is that it was reproducible across two different hospitals including a large university-affiliated and smaller academic community hospital. A limitation is that both hospitals have a low prevalence of VRE and it is not known whether NPV would be lower in hospitals with higher prevalence. The results should not be applied beyond point prevalence testing for VRE.

While further external validation is needed, antibiotic-exposure guided prevalence screening for VRE has significant potential to contribute to improved resource stewardship.
